# Editorial: Recent advancements in modeling and simulations of ion channels

**DOI:** 10.3389/fmolb.2022.1098216

**Published:** 2022-11-22

**Authors:** Simone Furini, Luca Maragliano, Matteo Masetti

**Affiliations:** ^1^ Department of Electrical, Electronic and Information Engineering “Guglielmo Marconi”, Alma Mater Studiorum–University of Bologna, Cesena, Italy; ^2^ Department of Life and Environmental Sciences, Polytechnic University of Marche, Ancona, Italy; ^3^ Center for Synaptic Neuroscience and Technology (NSYN@UniGe), Istituto Italiano di Tecnologia, Genova, Italy; ^4^ Department of Pharmacy and Biotechnology, Alma Mater Studiorum–University of Bologna, Bologna, Italy

**Keywords:** molecular dynamics simulation, enhanced sampling algorithms, multiscale modeling, channel gating mechanism, ion conduction

Computational modeling and simulations have long been recognized as key tools for a detailed characterization of ion channel functionality, which is pivotal for a deeper understanding of several physiological and pathophysiological processes. This is particularly relevant in light of the currently available computational resources that finally allow studying the complex molecular mechanisms driving conduction, selectivity, and gating. Still, the need to provide a direct link with experimental measurements calls for advanced simulation frameworks, including enhanced sampling techniques and/or multiscale models, while adequate analysis tools must be devised to cope with an ever-increasing amount of data. Articles on this Research Topic reflect the close interplay among these aspects and provide insight into advances in computational simulations in the field of ion channels both from a methodological and application standpoint.

Gating and allosteric activation of the Orai protein belonging to the calcium-release activated calcium (CRAC) channel family is an example of complex molecular mechanisms that can be untangled through computational approaches. By performing repeated sub-microsecond-long molecular dynamics (MD) simulations based on the Orai crystal structure from *Drosophila melanogaster* (dOrai), Zhang et al. showed that increased pore hydration in the gain-of-function L210F mutant facilitates channel opening with respect to the wildtype (WT) channel, regardless of activator binding. The dOrai gating mechanism was instead explicitly investigated through enhanced sampling by Guardiani et al. A complex allosteric propagation mechanism going from the activator binding site to the inner helices was revealed through contact map analysis, which was recapitulated into a gating mechanism involving a “steric brake” and the formation of vacuum bubbles inside the pore. Contact map analysis, complemented with network analysis, was also exploited by Costa et al. for highlighting the molecular determinants underlying the inactivation mechanism of the voltage-gated potassium channel K_v_1.2. The approach allowed the authors to identify two distinct allosteric pathways coupling the voltage-sensing domain and the pore domain to the selectivity filter, where channel inactivation is expected to occur. The allosteric pathways were compared with those obtained from nine mutants known to impair the inactivation mechanism, supporting their hypothesis.

The system size required to correctly describe the molecular assemblies under investigation is critical to consider when facing ion channel modeling. For studying the claudin-15 strands and their role in tight junctions, Fuladi et al. adopted a combination of all-atom and hybrid resolution schemes, which allowed them to simulate double-membrane systems up to the sub-micrometer length scale. By comparing the dynamics of the WT and A134P mutant, the authors showed that the mutation significantly affects the mechanical properties of the strands, including lateral flexibility and persistence length. Rationalizing the impact of mutations on the dynamics and function of a member of the superfamily of pentameric ligand-gated ion channels (PLGICs), the Glycine Receptor (GlyR), was instead the subject of the contribution by Mhashal et al. Through the integration of bioinformatics tools and structural analysis, the authors identified three mutations reported in human tumors linked to the disruption of glycinergic currents that were expected to impact GlyR conformation. Then, by mapping the MD trajectories on a low-dimensionality space capturing the conformational variability encoded in several functional states of the channel, they highlighted a divergent behavior of the mutants with respect to the WT. This example emphasizes the role played by sophisticated analysis tools both in planning and interpreting simulation results. In this context, Raffo et al. presented a novel analysis method for the geometrical characterization of the shape and dynamics of ion channels which does not rely on user-dependent parameters, like the pore axis, and that is specifically devised for processing MD trajectories.

Modulation of channels’ dynamics and functionality through ligand binding is another aspect of ion channel research that can benefit from advances in computational simulations. For example, Garofalo et al. characterized the binding mode of the small-molecule modulator retigabine to the K_v_7.2 channel through an elaborate computational framework involving homology modeling, MD simulations, and ensemble docking. Specifically, they showed that retigabine can bind multiple functional states of the channel and provided molecular insights into the ligand-induced activation process. The importance of channels as pharmaceutical targets was also underscored by Aledavood et al. In their Review, the authors provide a comprehensive picture of the structural and mechanistic aspects related to the function of the M2 proton channel of the Influenza A virus, and how this knowledge can be exploited for computer-aided drug design, especially in light of the emergence of resistant strains.

Ion permeation is perhaps the most distinctive feature of ion channel functionality that poses specific challenges to modeling and simulations. Lin and Luo presented a systematic benchmark of widespread approaches to single-channel permeability calculation applied on a carbon nanotube as a small-conductance ion channel model. Comparing results obtained from enhanced sampling methods like umbrella sampling and milestoning on the one hand, and out-of-equilibrium steady-state flux under applied voltage on the other, they discuss the advantages and drawbacks of the different techniques, providing useful guidelines for further investigations in the field. Finally, a bottom-up multiscale approach was developed by Horng et al. to study the longtime controversial mechanism of K^+^ permeation through the prototypical KcsA channel. The proposed method is based on a kinetic model fed with rate constants obtained from enhanced sampling. In this way, they managed to estimate current-voltage and current-concentration characteristics, providing a link between atomic structures and single-channel experimental data.

Overall, the studies presented in this Research Topic demonstrate the liveliness of computational methods in the field of ion channels and how methodological advances in simulations and analysis frameworks are fostering a better understanding of the molecular mechanisms responsible for their functionality.

